# Author Correction: Hexafluorophosphate additive enables durable seawater oxidation at ampere-level current density

**DOI:** 10.1038/s41467-025-61100-w

**Published:** 2025-06-19

**Authors:** Xun He, Yongchao Yao, Limei Zhang, Hefeng Wang, Hong Tang, Wenlong Jiang, Yuchun Ren, Jue Nan, Yongsong Luo, Tongwei Wu, Fengming Luo, Bo Tang, Xuping Sun

**Affiliations:** 1 Center for High Altitude Medicine, West China Hospital, Sichuan University, Chengdu, Sichuan China; 2 Institute of Fundamental and Frontier Sciences, University of Electronic Science and Technology of China, Chengdu, Sichuan China; 3 College of Chemistry, Chemical Engineering and Materials Science, Shandong Normal University, Jinan, Shandong China; 4 Department of Laboratory Medicine/Clinical Laboratory Medicine Research Center, West China Hospital, Sichuan University, Chengdu, Sichuan China; 5 Laoshan Laboratory, Qingdao, Shandong China

**Keywords:** Electrocatalysis, Hydrogen energy, Electrocatalysis, Electrocatalysis

Correction to: *Nature Communications* 10.1038/s41467-025-60413-0, published online 29 May 2025

In the version of the article initially published, there were errors in Fig. 4c and Supplementary Fig. 34c where the labels “NiFe LDH/NF||Pt/C/NF” originally appeared as “NiFe LDH/NF (-) || Pt/C/NF (+).” The figures have been corrected in the HTML and PDF versions of the article.

