# Supplementary material for: Hexafluorophosphate additive enables durable seawater oxidation at ampere-level current density
Source: Nat Commun. 2025 May 29;16:4998. doi: 10.1038/s41467-025-60413-0 (PMC12122868; doi:10.1038/s41467-025-60413-0)
Supplement: Supplementary file 1 — Supplementary Information [file 41467_2025_60413_MOESM1_ESM.pdf]

Supplementary Information

Hexafluorophosphate additive enables durable seawater oxidation at ampere-level current density

Xun He[1,2,3,#], Yongchao Yao[1,4,#], Limei Zhang[2,4], Hefeng Wang[3], Hong Tang[2], Wenlong Jiang[2], Yuchun Ren[2], Jue Nan[2], Yongsong Luo[1], Tongwei Wu[2*], Fengming Luo[1*], Bo Tang[3,5*] & Xuping Sun[1,3*]

[1]Center for High Altitude Medicine, West China Hospital, Sichuan University, Chengdu 610041, Sichuan, China. [2]Institute of Fundamental and Frontier Sciences, University of Electronic Science and Technology of China, Chengdu 610054, Sichuan, China. [3]College of Chemistry, Chemical Engineering and Materials Science, Shandong Normal University, Jinan 250014, Shandong, China. [4]Department of Laboratory Medicine/Clinical Laboratory Medicine Research Center, West China Hospital, Sichuan University, Chengdu 610041, Sichuan, China. [5]Laoshan Laboratory, Qingdao 266237, Shandong, China. [#]Both authors contributed equally to this work.

[*]Correspondence and requests for materials should be addressed to T.W. (email: twwu77@uestc.edu.cn) or F.L. (email: luofengming@wchscu.edu.cn) or B.T. (email: tangb@sdnu.edu.cn) or X.S. (email: xpsun@uestc.edu.cn).

Supplementary Note 1: Chloride corrosion mechanism on electrode materials.

Chloride corrosion occurs in a sequence of three main steps: polarization, dissolution, and hydrolysis.

1. Adsorption of Cl^- through surface polarization.

The process begins with the adsorption of Cl^- onto the electrode surface, leading to the following reaction:

$M + Cl^- \rightarrow MCl_{ads}^{x-1} + xe^-$

2. Dissolution through further coordination.

Following adsorption, the chloride complex undergoes further coordination, where the adsorbed chloride interacts with additional Cl^-, leading to dissolution of the metal:

$MCl_{ads}^{x-1} + (x\text{-}1)\ Cl^- \rightarrow MCl_x^-$:

3. Conversion from chloride to hydroxide.

Finally, the chloride species reacts with OH^- in an aqueous environment, leading to the conversion of the metal chloride to metal hydroxide:

$MCl_x^- + xOH^- \rightarrow M(OH)_x + xCl^-$.

Supplementary Note 2: Estimation of the energy consumption and hydrogen production cost of the NiFe LDH/NF||Pt/C/NF flow-type electrolyzer.

The NiFe LDH/NF||Pt/C/NF flow-type electrolyzer operated under a dual-feed configuration using 6 M KOH + seawater as both the anolyte and catholyte, with PF_6^- introduced into the anolyte. The energy consumption was evaluated under electrolysis conditions of 1.0 A cm^{-2}, 2.02 V, 60 °C, and an electrode area of 1 × 1 cm^2. Under these conditions, the power input was calculated to be 2.02 W cm^{-2}. The hydrogen production rate was determined to be 5.18×10^{-6} mol cm^{-2} s^{-1}. Taking the lower heating value (LHV) of H_2 as 120 MJ kg^{-1} (equivalent to 241.9 kJ mol^{-1}), the output power density associated with H_2 generation was estimated to be 1.253 W cm^{-2}. Accordingly, the energy efficiency (based on LHV) was calculated to be 62.0%. The electricity consumption per kilogram of hydrogen was further estimated as 53.7 kWh kg^{-1} H_2 (4.49 kWh m^{-3}), corresponding to a production cost of US$1.07 kg^{-1} H_2, when utilizing offshore renewable electricity at US$0.02 kWh^{-1}.

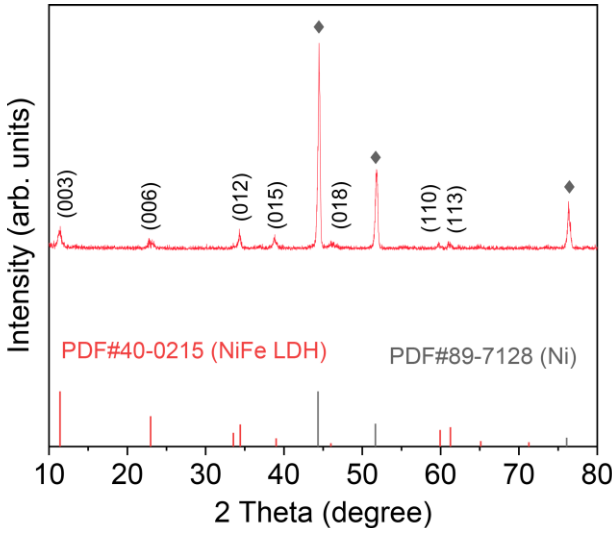

Supplementary Fig. 1 | XRD pattern. XRD pattern of NiFe LDH/NF. Source data are provided as a Source Data file.

XRD pattern analysis verifies the NiFe LDH on Ni, with diffraction peaks matching the lattice planes of NiFe LDH (JCPDS#40-0215) and metallic Ni (JCPDS#89-7128).

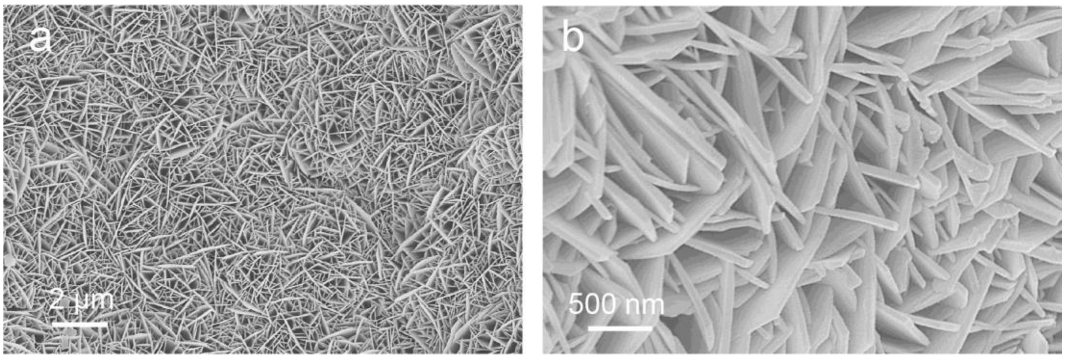

Supplementary Fig. 2 | SEM images. (a) Low- and **(b)** high-magnification SEM images of NiFe LDH/NF.

SEM analysis displays the 2D nanosheet array structure of the prepared NiFe LDH. Such structures have been extensively reported to possess strong hydrophilicity, thus promoting efficient mass transfer of O_2 during electrolysis and offering a certain degree of mechanical buffering against external forces (*Adv. Mater.* **36**, 2311322 (2024); *Acc. Chem. Res.* **51**, 1590 (2019); *Adv. Mater.* **27**, 2361 (2015); *Nat. Commun.* **15**, 4712 (2024); *ACS Catal*. **11**, 13140 (2021)).

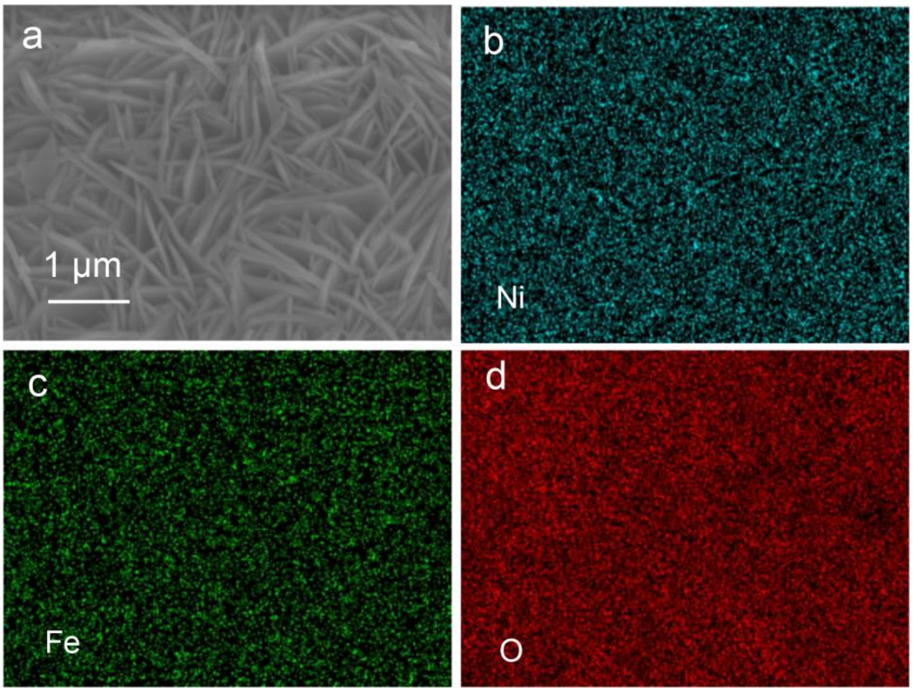

Supplementary Fig. 3 | Elemental distributions. (a) SEM image and its corresponding energy-dispersive X-ray spectroscopy (EDS) images **(b–d)** of NiFe LDH/NF.

SEM and EDS analyses reveal the uniform distribution of Ni, Fe, and O on NiFe LDH/NF.

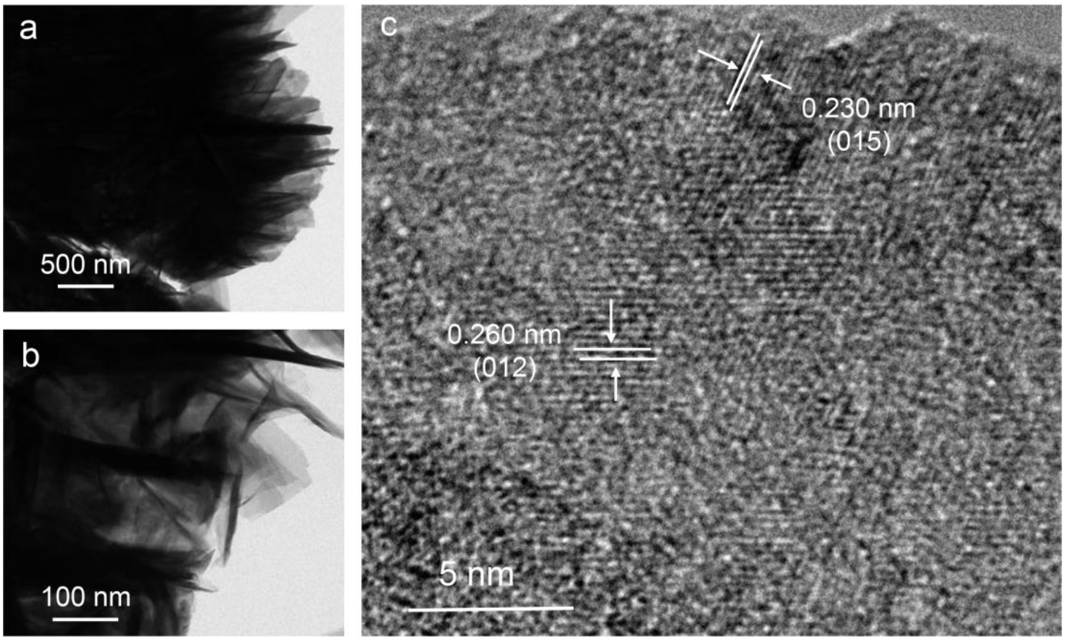

Supplementary Fig. 4 | TEM images and HRTEM image. (a) Low- and **(b)** high-magnification TEM images of NiFe LDH. **(c)** HRTEM image of NiFe LDH.

As depicted in Supplementary Fig. 4a, b, the sample further demonstrates the characteristic 2D nanosheet structure of LDH. HRTEM analysis (Supplementary Fig. 4c) confirms lattice spacings of 0.230 nm and 0.260 nm, corresponding to the (015) and (012) crystallographic planes of NiFe LDH, respectively.

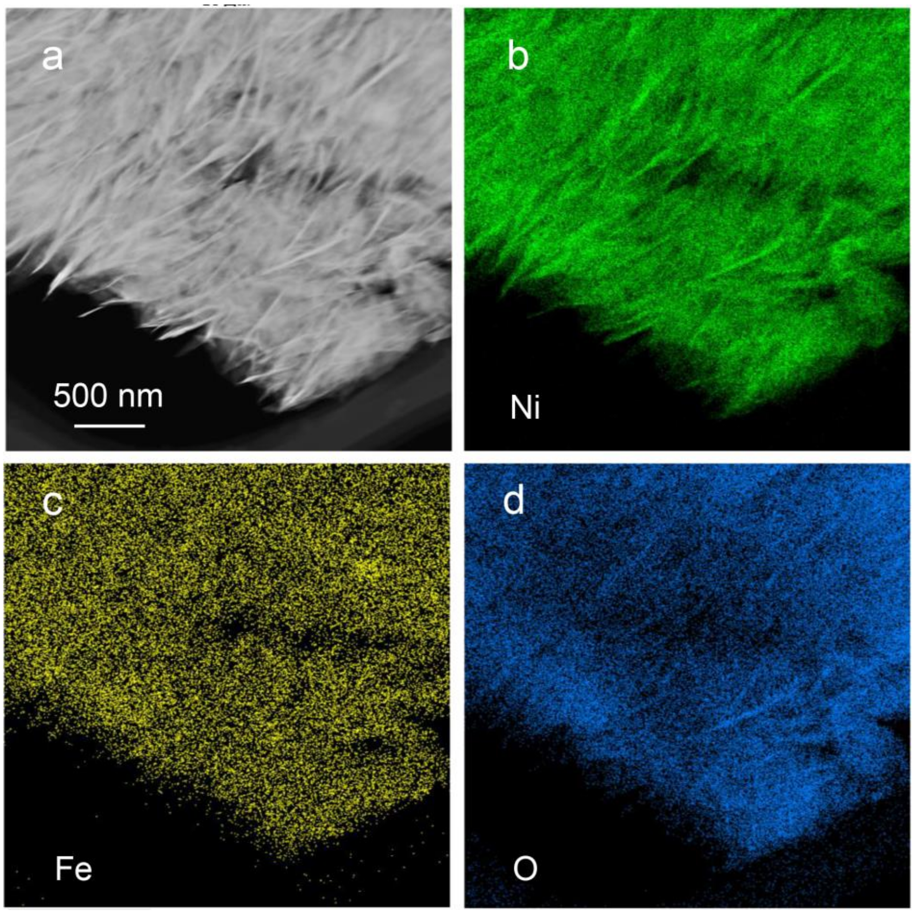

Supplementary Fig. 5 | Elemental distributions. (a) High-angle annular dark field scanning transmission electron microscopy (HAADF-STEM) image and **(b-d)** its corresponding EDS elemental mapping images of NiFe LDH.

The use of HAADF-STEM and EDS elemental mapping confirms that Ni, Fe, and O elements are uniformly dispersed throughout the 2D LDH nanosheets.

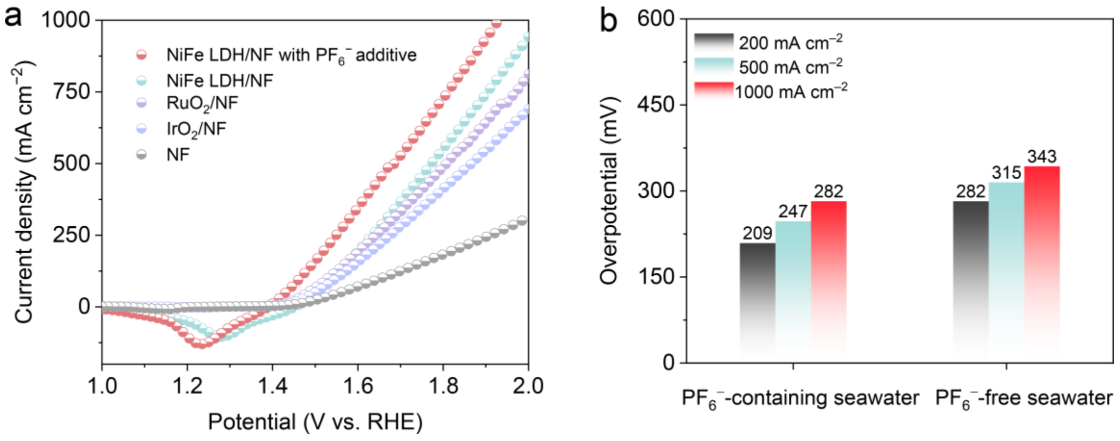

Supplementary Fig. 6 | Evaluation of activities. **(a)** Polarization curves of NiFe LDH/NF in PF_6^--free and PF_6^--containing seawater, alongside RuO_2/NF, IrO_2/NF and NF in PF_6^--free seawater without *iR* compensation. The corresponding solution resistances are 1.700± 0.03 Ω and 1.544 ± 0.01 Ω for NiFe LDH/NF, and 1.767± 0.02 Ω, 1.762± 0.02 Ω, and 1.757± 0.05 Ω for RuO_2/NF, IrO_2/NF, and NF, respectively. The area of all electrodes is 0.25 cm^{-2}. (**b**) Comparison of overpotentials for NiFe LDH/NF in PF_6^--free and PF_6^--containing seawater with 100% *iR* correction. Source data are provided as a Source Data file.

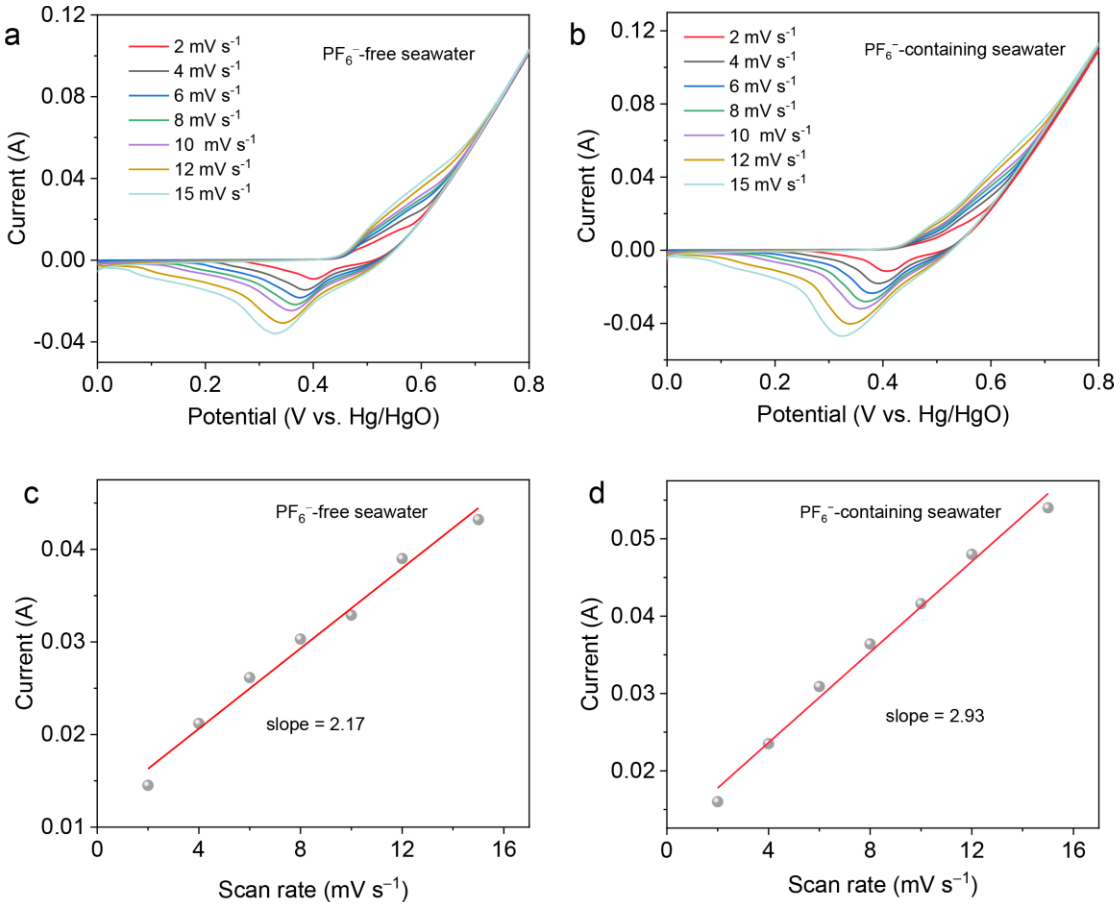

Supplementary Fig. 7 | Evaluation of activities. CV curves of NiFe LDH/NF in **(a)** PF_6^--free and **(b)** PF_6^--containing seawater and corresponding relationships between oxidation peak currents and scan rates of NiFe LDH/NF in **(c)** PF_6^--free and **(d)** PF_6^--containing seawater, respectively. Slopes of 2.17 ± 0.01 and 2.93 ± 0.03 were obtained from triplicate tests in PF_6^--free and PF_6^--containing seawater, respectively. Only representative data are shown. Source data are provided as a Source Data file.

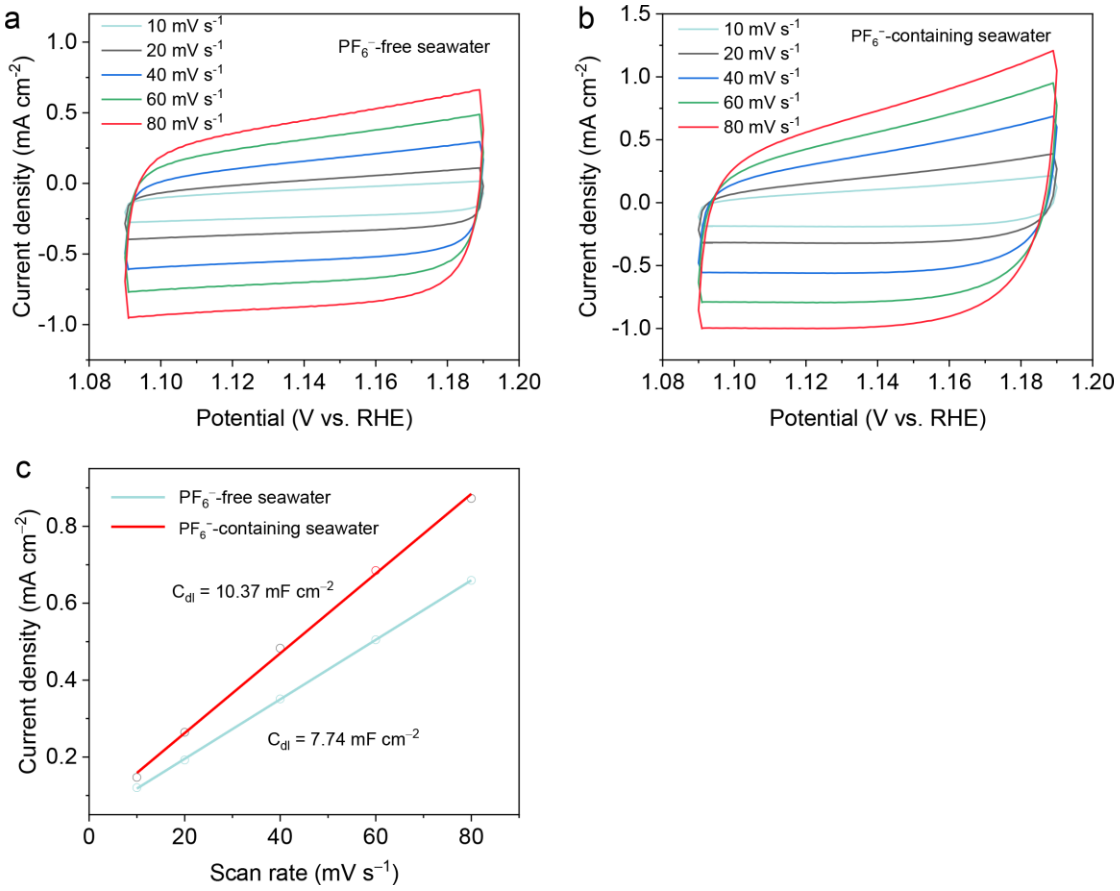

Supplementary Fig. 8 | Electrochemical double-layer capacities (C_{dl}) measurements. CV curves in the non-Faradaic zone at various scan rates for NiFe LDH/NF in **(a)** PF_6^--free and **(b)** PF_6^--containing seawater without *iR* correction. **(c)** Comparison of the C_{dl} values for NiFe LDH/NF in PF_6^--free and PF_6^--containing seawater. Source data are provided as a Source Data file.

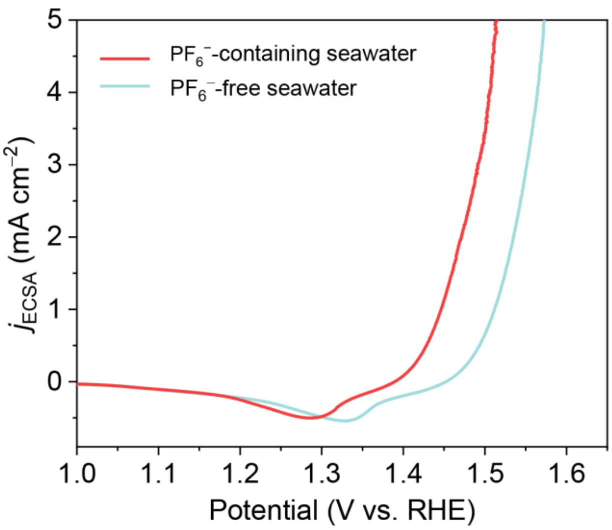

Supplementary Fig. 9 | Evaluation of activities. Polarization curves of NiFe LDH/NF in PF_6^--free and PF_6^--containing seawater with ECSA fitting with 100% *iR* correction. Source data are provided as a Source Data file.

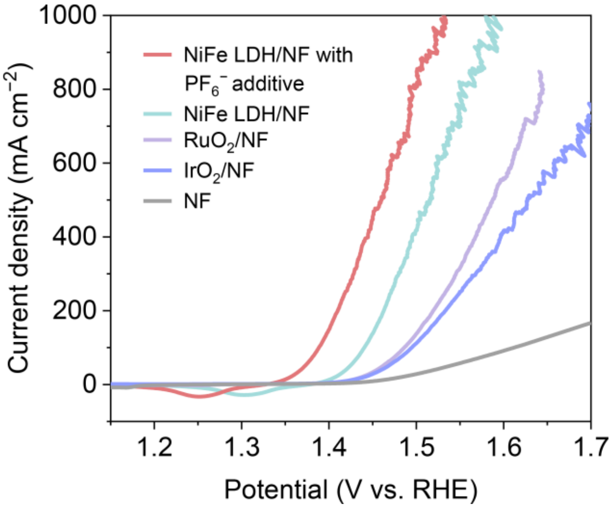

Supplementary Fig. 10 | Evaluation of activities. Polarization curves of NiFe LDH/NF in PF_6^--free and PF_6^--containing seawater, along with RuO_2/NF, IrO_2/NF, and NF in PF_6^--free seawater with 100% *iR* correction, measured at a slow scan rate of 1 mV s^{-1}. This slow scan rate was employed to avoid interference from non-OER chemical processes to ensure reliable determination of the Tafel slope. Source data are provided as a Source Data file.

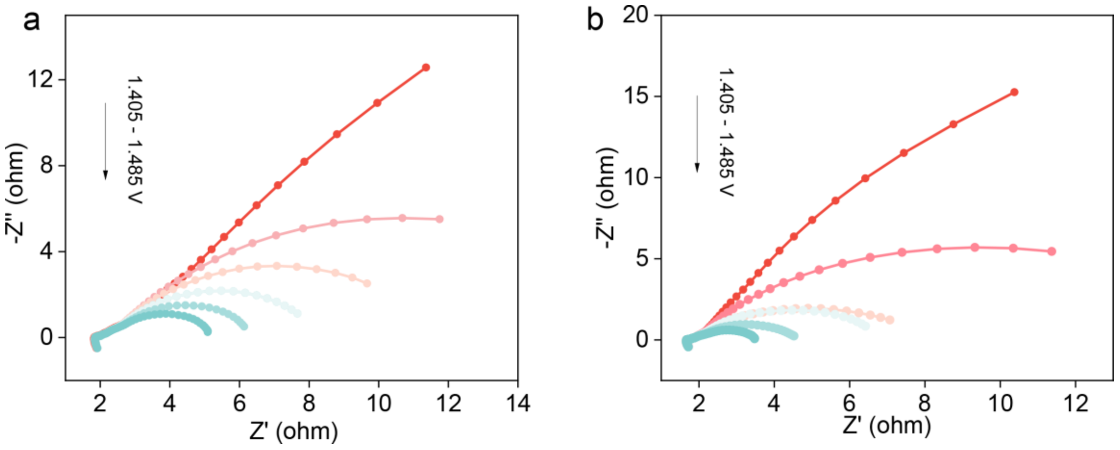

Supplementary Fig. 11 | Evaluation of kinetics. Electrochemical impedance spectroscopy (EIS) Nyquist plots collected from 1.405 to 1.485 V (1.405, 1.415, 1.435, 1.455, 1.475, 1.485 V) over a frequency range of 10^{-2} to 10^{5} Hz for NiFe LDH/NF in **(a)** PF_6^--free and **(b)** PF_6^--containing seawater. Source data are provided as a Source Data file.

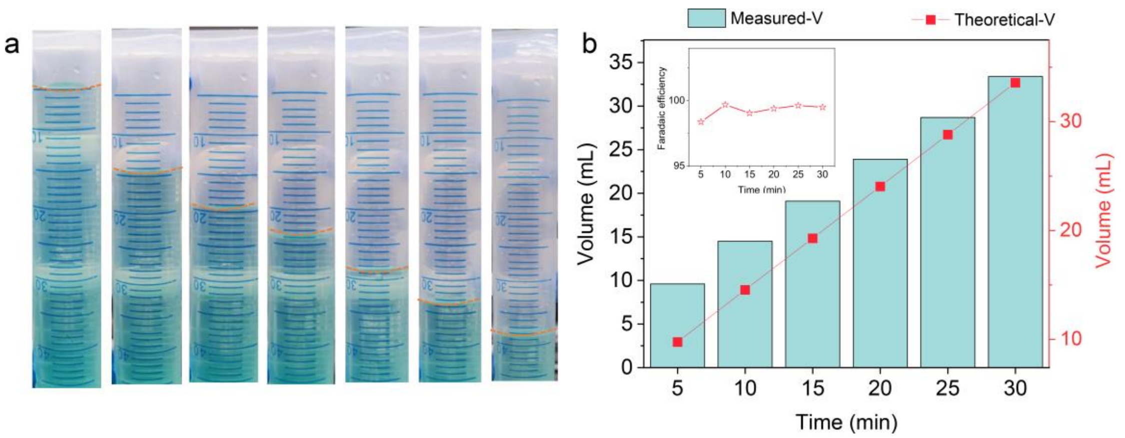

Supplementary Fig. 12 | O_2 Faradaic efficiency measurements in PF_6^--containing seawater. (a) Digital photographs of the collected O_2 during ASO. **(b)** Comparison of collected O_2 with theoretical values for NiFe LDH/NF in PF_6^--containing seawater at 1 A cm^{-2}. Source data are provided as a Source Data file.

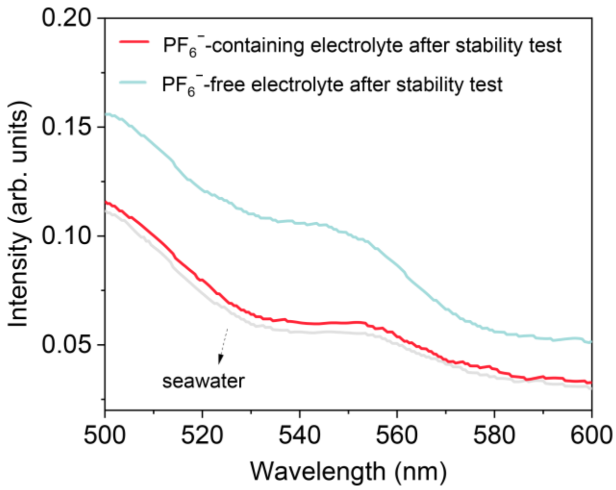

Supplementary Fig. 13 | Chlorine precipitation tests. Ultraviolet–visible (UV–vis) spectra of the ClO^- concentrations for the blank electrolyte and PF_6^--free and PF_6^--containing electrolyte for NiFe LDH/NF after the stability test at 1 A cm^{-2}. Source data are provided as a Source Data file.

We determined active chlorine concentrations by employing the DPD colorimetric method (*J. Electroanal. Chem*. **819**, 260–268 (2018); *Nano Today* **58**, 102454 (2024)) in conjunction with UV–vis spectroscopy. After operating the stability tests at 1 A cm^{-2}, 100 µL of the electrolyte was combined with 50 µL of both 1.0 M H_2SO_4 and 2.0 M NaOH, followed by 4.8 mL of deionized water. Subsequently, 250 µL of DPD reagent and phosphate-buffered saline (*pH*=6.5) were introduced. This mixture developed a pink hue, indicative of chlorine, which was then detected at 550 nm.

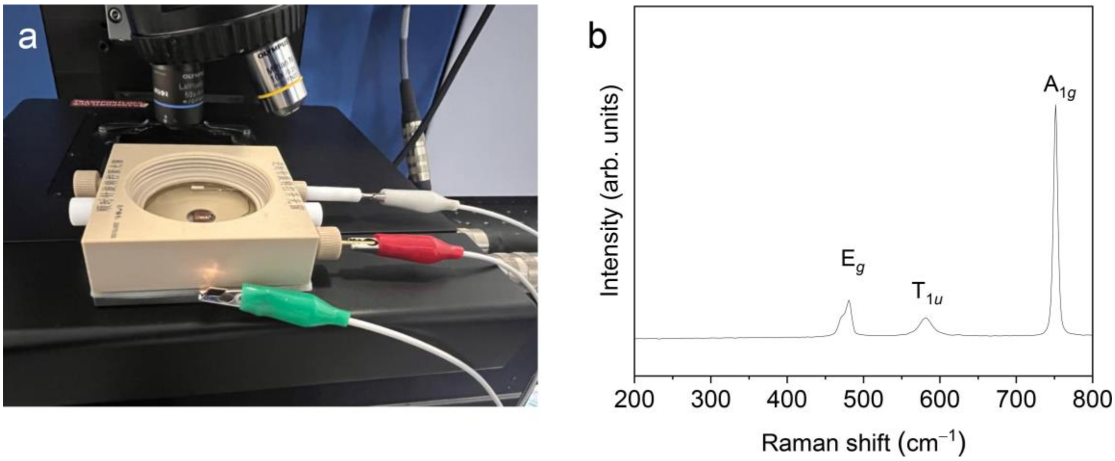

Supplementary Fig. 14 | Raman spectrum. (a) The photo of electrochemical cell for in situ Raman test. **(b)** Raman spectrum of KPF_6. Source data are provided as a Source Data file.

The Raman peaks at 480, 581, and 752 cm^{-1} are assigned to the E_g, T_{1u}, and A_{1g} vibrational modes of PF_6^-, respectively. The A_{1g} vibration of PF_6^- represents a fully symmetric stretching vibration in which all P–F bonds simultaneously lengthen or shorten. The E_g vibration is a doubly degenerate bending vibration, typically described as a combination of bond stretching and contraction across opposing P–F bonds (*J. Fluorine Chem*. **101**, 173–179 (2000); *J. Mol. Struct*. **1026**, 145–149 (2012); *Spectrochim. Acta A* **153**, 651–654 (2016)). Under intercalation conditions, the E_g bending mode becomes discernible due to confinement of PF_6^- within the interlayers, where the local coordination environment partially breaks the octahedral symmetry and modifies the polarizability change associated with bending vibrations. The A_{1g} symmetric stretching mode is weak or nearly absent in this intercalated state, presumably because PF_6^- is dispersed and partially disordered between the layers. As the applied potential increases, PF_6^- gradually accumulates on the electrode surface driven by a higher applied electric field, thereby enhancing the A_{1g} response.

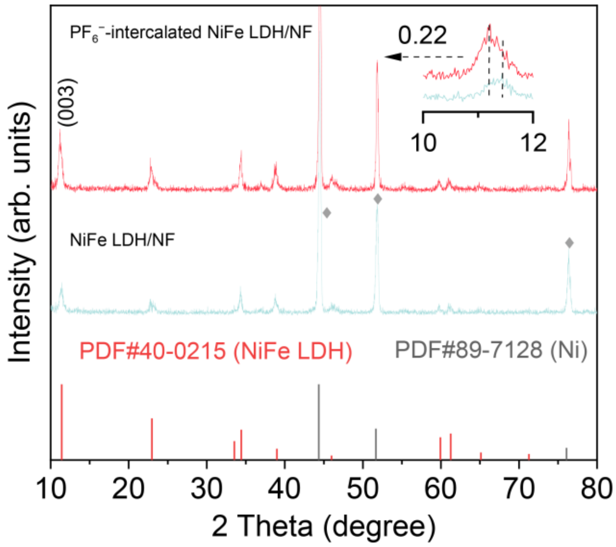

Supplementary Fig. 15 | XRD patterns. Comparison of the XRD patterns of NiFe LDH/NF before and after being held at 1.15 V for 10 min in PF_6^--containing seawater. Source data are provided as a Source Data file.

The (003) diffraction peak shifts to lower 2θ values after the treatment, signifying an increase in interlayer spacing of NiFe LDH.

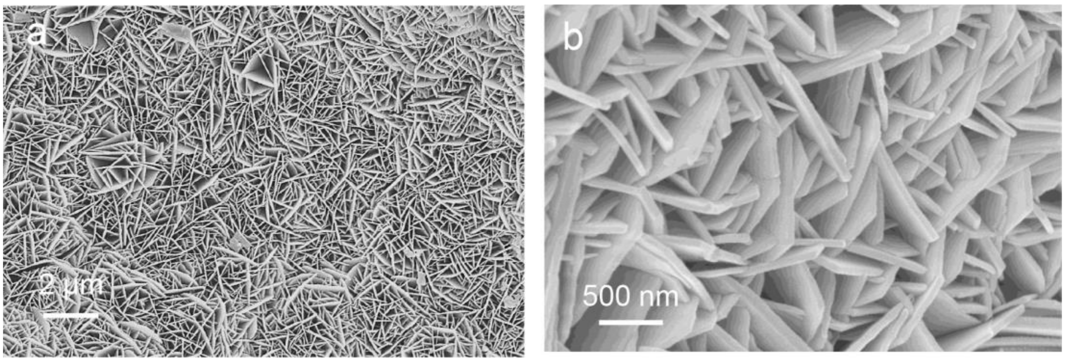

Supplementary Fig. 16 | SEM images. **(a)** Low- and **(b)** high-magnification SEM images of NiFe LDH/NF after being held at 1.15 V for 10 min in PF_6^--containing seawater.

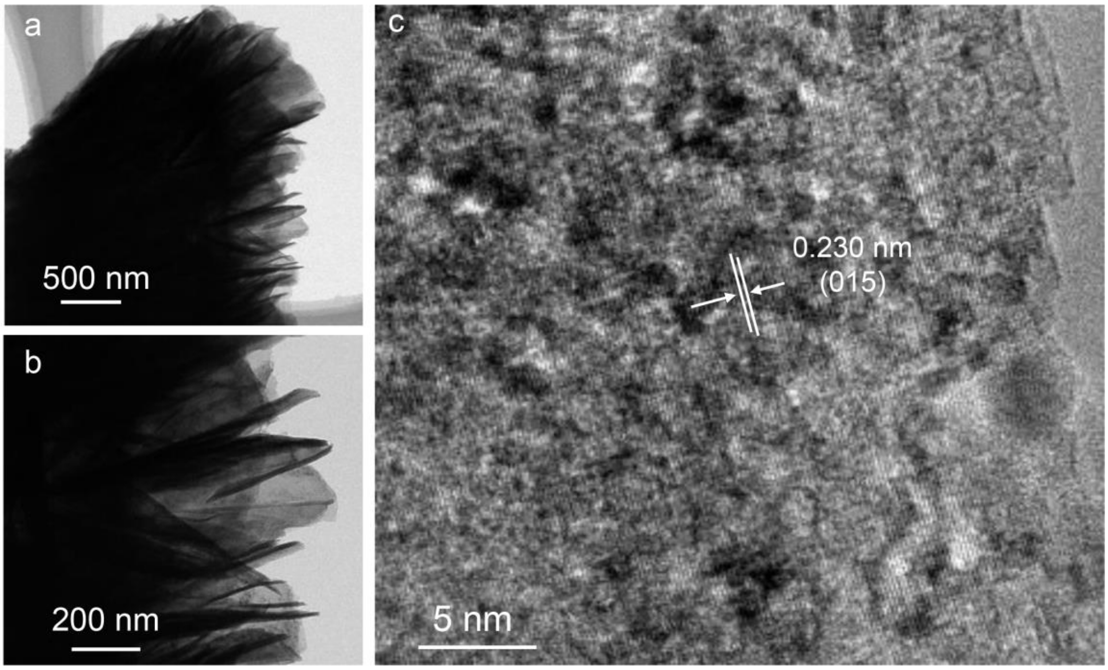

Supplementary Fig. 17 | TEM images and HRTEM image. **(a)** Low- and **(a)** high-magnification TEM images of NiFe LDH after being held at 1.15 V for 10 min in PF_6^--containing seawater. **(c)** HRTEM image of NiFe LDH after being held at 1.15 V for 10 min in PF_6^--containing seawater.

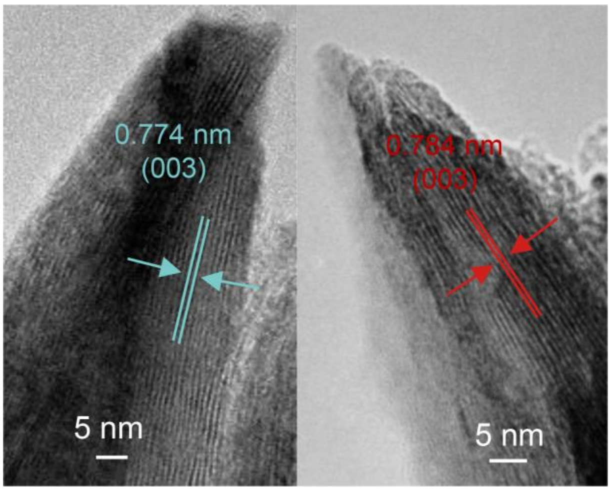

Supplementary Fig. 18 | HRTEM images. HRTEM images of NiFe LDH before (left) and after (right) being held at 1.15 V for 10 min in PF_6^--containing seawater.

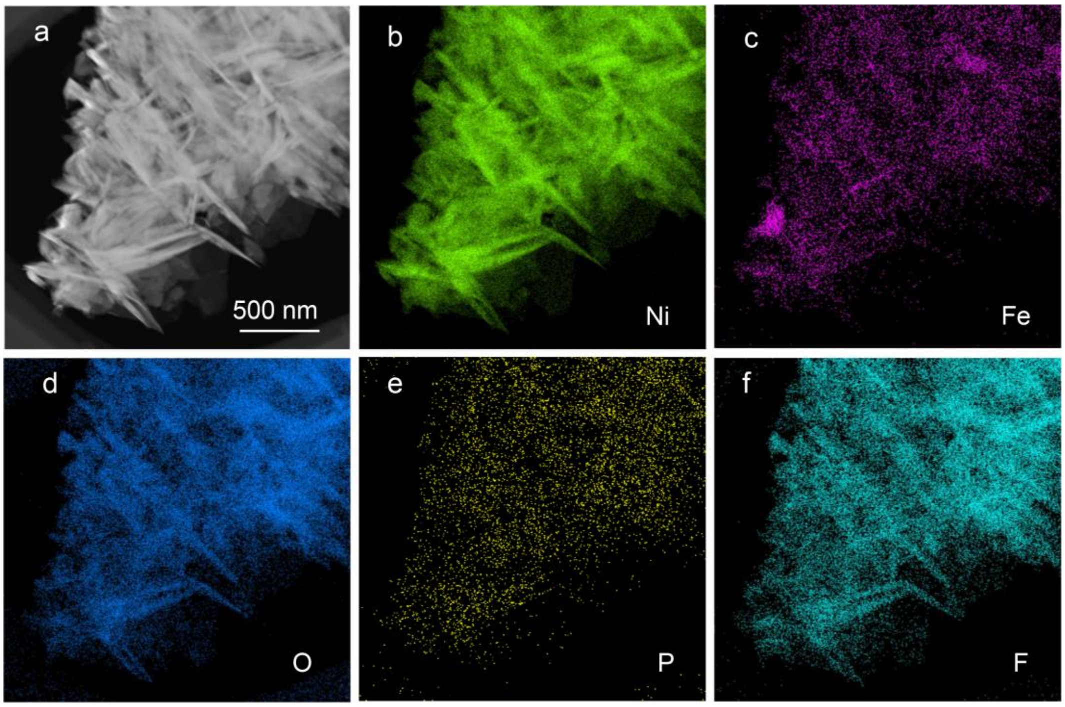

Supplementary Fig. 19 | Elemental distributions. (a) HAADF-STEM image and **(b–f)** corresponding elemental mapping images of NiFe LDH after being held at 1.15 V for 10 min in PF_6^--containing seawater.

As shown in Supplementary Figs. 16, 17a, b, and 19a, the NiFe LDH after being held at 1.15 V for 10 min in PF_6^--containing seawater retains the characteristic two-dimensional nanosheet morphology of pristine NiFe LDH. High-resolution TEM analysis further reveals a lattice spacing of 0.230 nm, corresponding to the (015) crystal planes of NiFe LDH (Supplementary Fig. 17c). Notably, NiFe LDH after being held at 1.15 V for 10 min in PF_6^--containing seawater results in an increase in the interlayer distance from 0.774 nm to 0.784 nm, as evidenced by HRTEM images (Supplementary Fig. 18). Moreover, HAADF-STEM image and its corresponding EDS elemental mapping images confirm the uniform distribution of P and F elements throughout the NiFe LDH nanosheets (Supplementary Fig. 19). These results collectively demonstrate the successful electrochemical intercalation of PF_6^- into the interlayer galleries of NiFe LDH under applied potential.

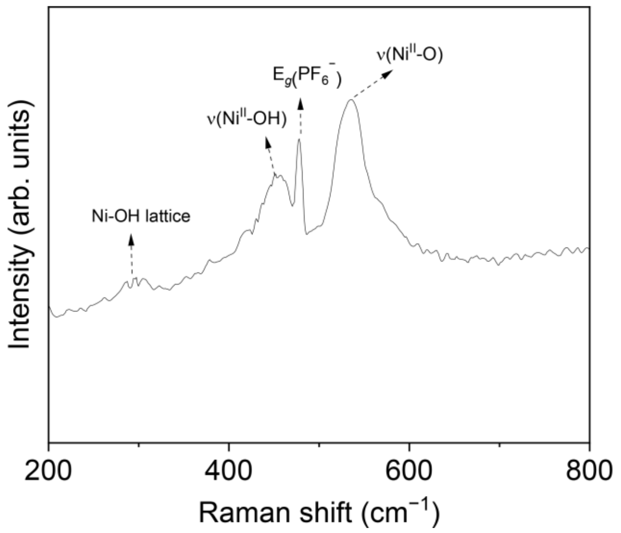

Supplementary Fig. 20 | Raman spectrum. Raman spectrum of PF_6^--intercalated NiFe LDH. Source data are provided as a Source Data file.

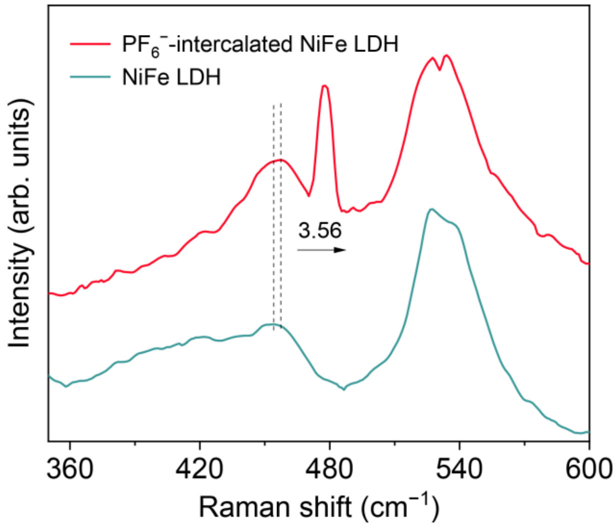

Supplementary Fig. 21 | Raman spectra. Comparison of Raman spectra of PF_6^--intercalated NiFe LDH and NiFe LDH at OCP. Source data are provided as a Source Data file.

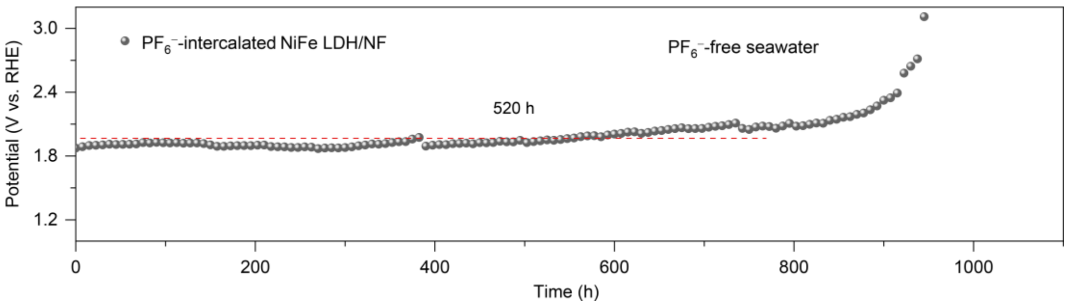

Supplementary Fig. 22 | Chronopotentiometry test. Chronopotentiometry curve operated at 2 A cm^{-2} for PF_6^--intercalated NiFe LDH/NF in PF_6^--free seawater without *iR* correction. Source data are provided as a Source Data file.

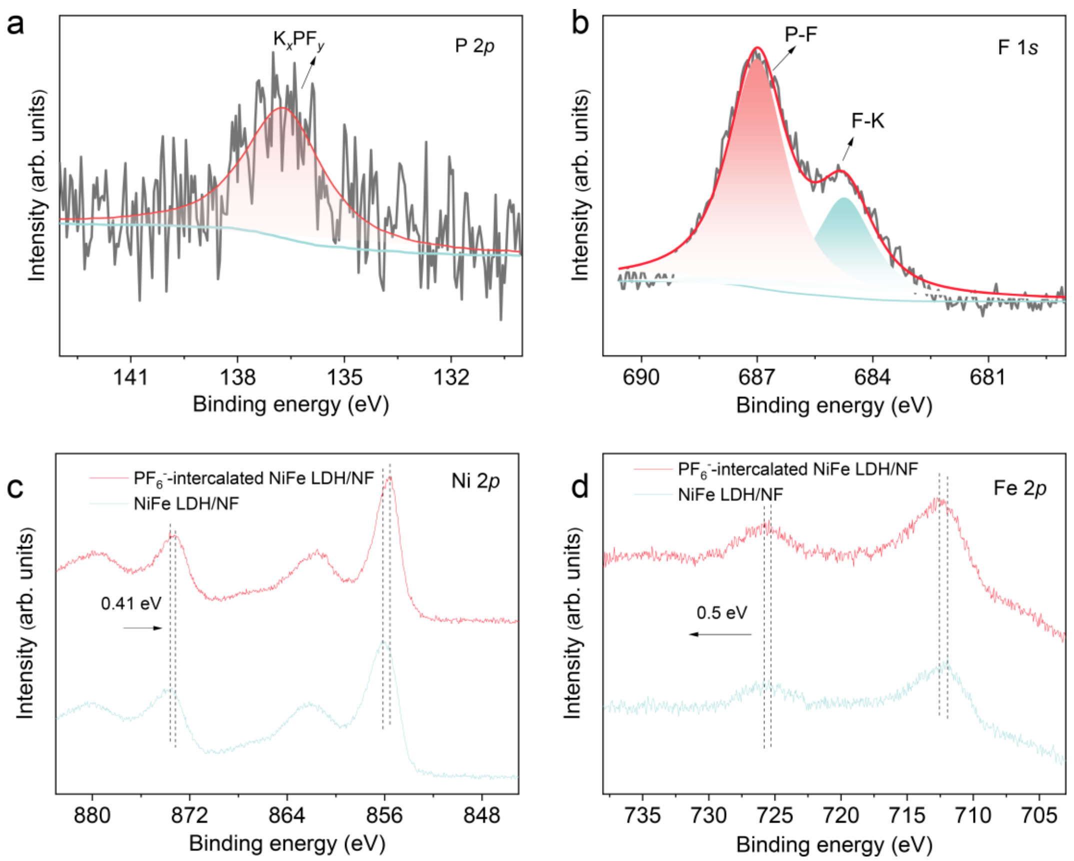

Supplementary Fig. 23 | XPS spectra. XPS spectra of PF_6^--intercalated NiFe LDH in the **(a)** P 2*p* and **(b)** F 1*s* regions. Comparison of XPS spectra of PF_6^--intercalated NiFe LDH and NiFe LDH in the **(c)** Ni 2 *p* and **(d)** Fe 2*p* regions. Source data are provided as a Source Data file.

The P 2*p* spectrum (Supplementary Fig. 23a) displays a peak at 136.7 eV, which is associated with the presence of K_xPF_y. Upon deconvolution, the F 1*s* XPS spectrum (Supplementary Fig. 23b) reveals two distinct peaks at 686.9 and 684.7 eV, corresponding to P–F and F–K, respectively (*ACS Appl. Mater. Interfaces* **11**, 22449–22456 (2019); *ACS Appl. Mater. Interfaces* **12**, 34873–34881 (2020)).

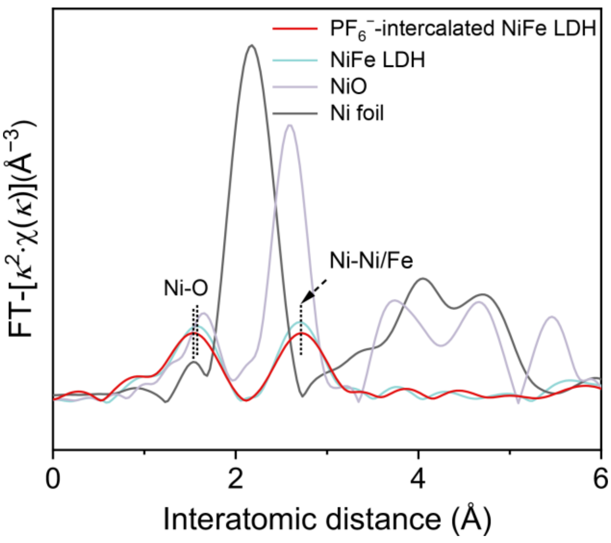

Supplementary Fig. 24 | First derivative Ni K-edge spectra of PF_6^--intercalated NiFe LDH and NiFe LDH, NiO, and Ni foil. Source data are provided as a Source Data file.

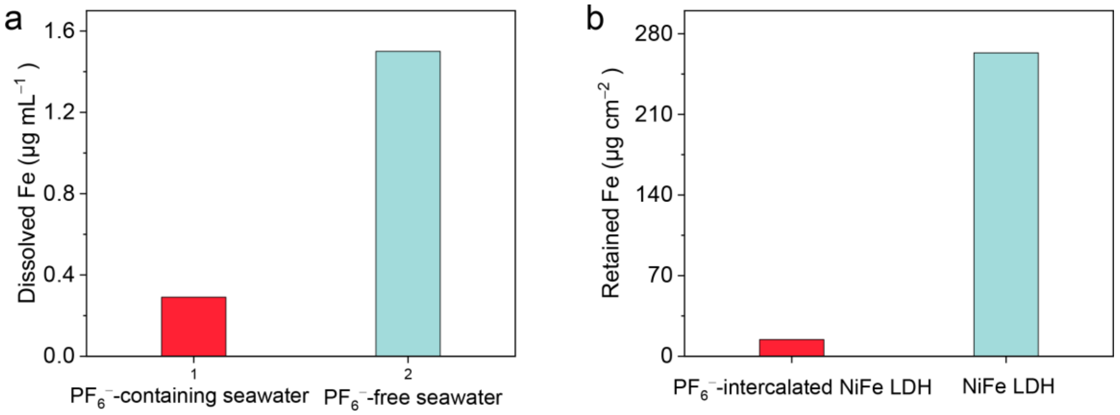

Supplementary Fig. 25 | (a) Evaluation of Fe ion leaching into electrolytes from NiFe LDH/NF electrodes in PF_6^--free and PF_6^--containing seawater after 120 hours of chronoamperometry tests at 1 A cm^{-2}. **(b)** Evaluation of retained Fe in PF_6^--intercalated NiFe LDH and NiFe LDH after 120 hours of chronoamperometry tests at 1 A cm^{-2}.

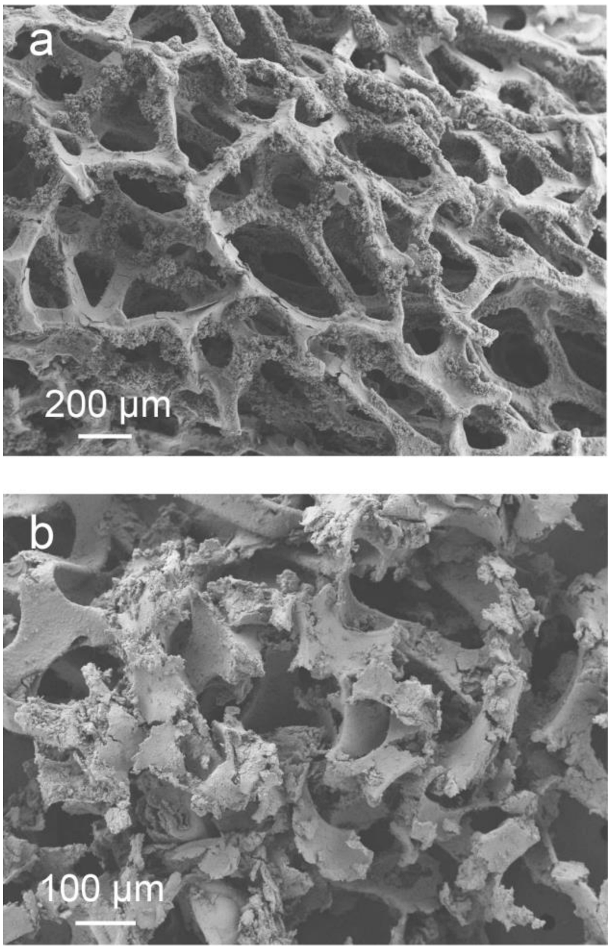

Supplementary Fig. 26 | SEM images. SEM images of NiFe LDH/NF after 120 h chronoamperometry tests at 1 A cm^{-2} in **(a)** PF_6^--containing and **(b)** PF_6^--free seawater.

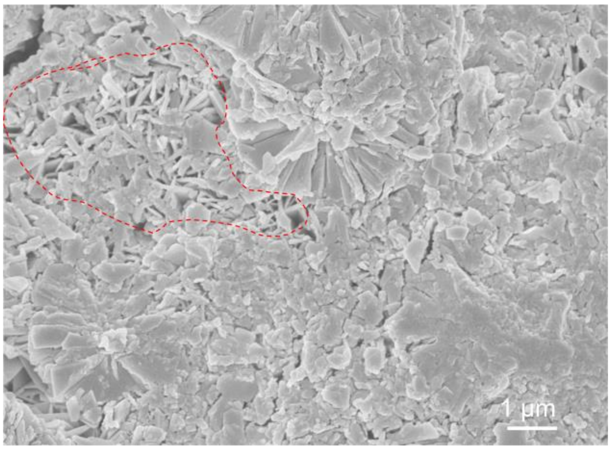

Supplementary Fig. 27 | SEM image of NiFe LDH/NF after stability test at 1 A cm^{-2} in PF_6^--containing seawater.

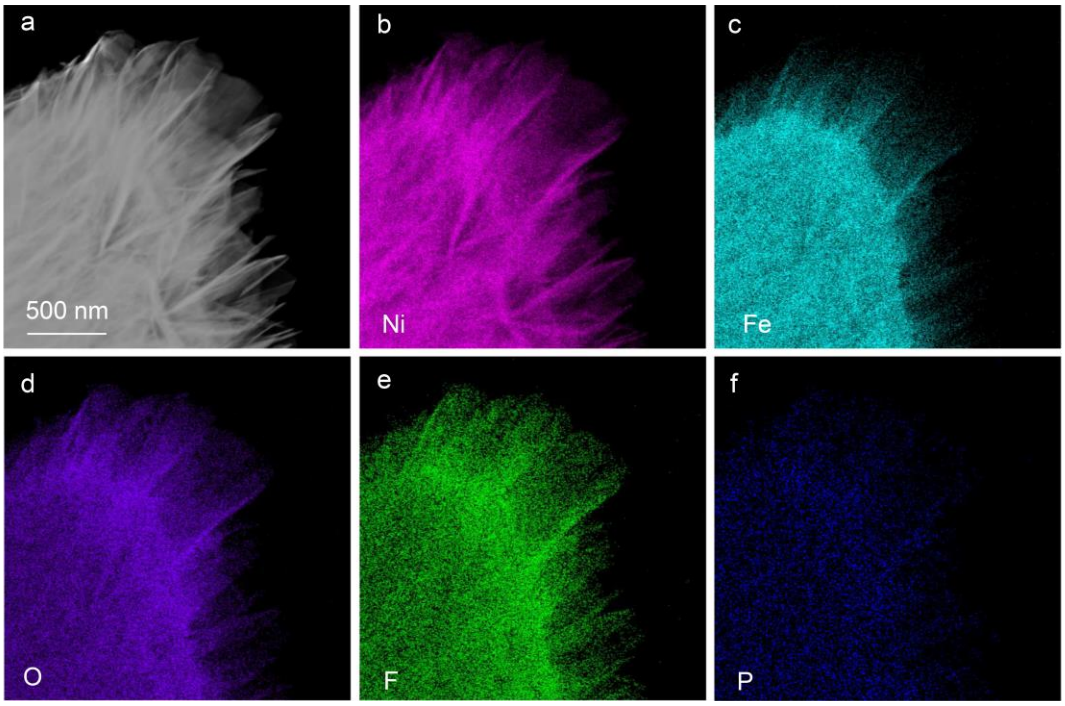

Supplementary Fig. 28 | Elemental distributions. (a) HAADF-STEM image and **(b–f)** corresponding elemental mapping images of NiFe LDH after stability test in PF_6^--containing seawater.

EDS analyses show the presence of P, F, Ni, Fe and O across the nanosheets, further demonstrating that PF_6^- incorporation stabilizes Fe and mitigates its leaching.

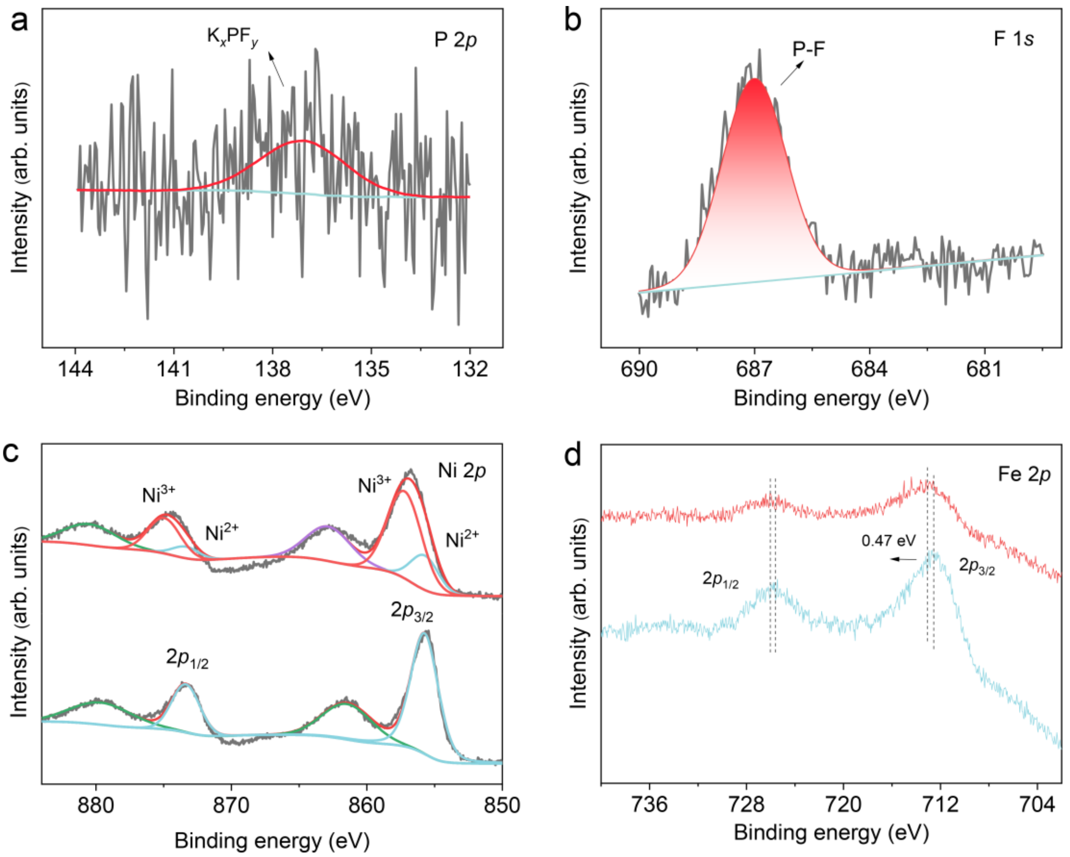

Supplementary Fig. 29 | XPS spectra. XPS spectra of NiFe LDH/NF in the **(a)** P 2*p* and **(b)** F 1*s* regions after stability test in PF_6^--containing seawater. XPS spectra of NiFe LDH/NF in the **(c)** Ni 2 *p* and **(d)** Fe 2*p* regions before and after stability test in PF_6^--containing seawater. Source data are provided as a Source Data file.

XPS analyses reveal that the P 2*p* and F 1*s* signals (Supplementary Fig. 29a, b) are relatively weak but consistent with those observed prior to electrolysis (Supplementary Fig. 23a, b), possibly due to partial deintercalation of PF_6^- from the interlayers under high potential. After long-term operation, the Ni 2*p* peak shifts to higher binding energy, indicating extensive surface oxidation from Ni^{2+} to Ni^{3+} (Supplementary Fig. 29c). Meanwhile, the Fe 2*p* spectrum shows only a minor shift without notable intensity loss (Supplementary Fig. 29d), also supporting effective stabilization of Fe by PF_6^- incorporation.

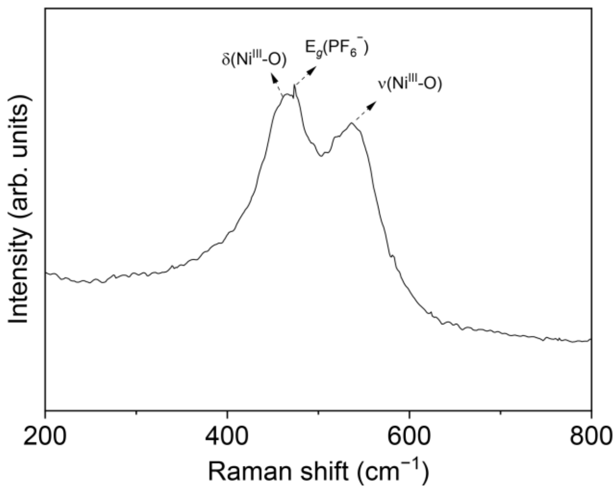

Supplementary Fig. 30 | Raman spectrum. Raman spectrum of NiFe LDH/NF after stability test in PF_6^--containing seawater. Source data are provided as a Source Data file.

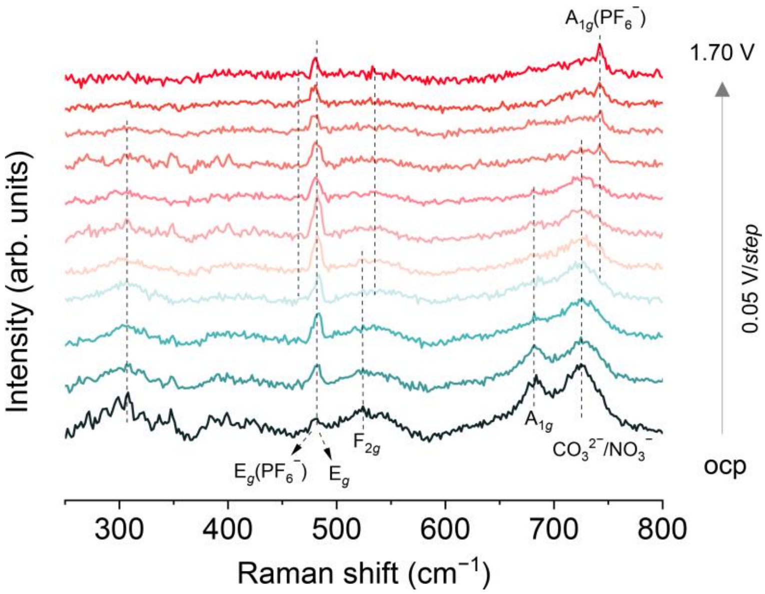

Supplementary Fig. 31 | Operando Raman spectra. Operando Raman spectra of CoFe LDH in PF_6^--containing seawater during ASO. Source data are provided as a Source Data file.

Operando Raman spectra of CoFe LDH in PF_6^--containing seawater were collected from OCP to 1.70 V vs. RHE (step: 0.05 V) to investigate PF_6^- intercalation and surface adsorption behaviors. The spectrum displays signals at 482, 523, and 698 cm^{-1}, corresponding to the E_g, F_{2g}, and A_{1g} vibrations of the symmetric stretching mode of Co–O and the bending mode of O–Co–O (*CrystEngComm* **24**, 6018–6030 (2022); *Chem. Mater*. **32**, 4303 (2020); *ACS Catal*. **8**, 1238–1247 (2018); *Chem. Eng. J*. **472**, 145076 (2023)). As the potential increases, the A_{1g} and F_{2g} bands and the Co–OH lattice signal at 300 cm^{-1} gradually vanish, and the shoulder for E_g and F_{2g} modes at 464 and 536 cm^{-1} appears (partially masked by PF_6^- signals), indicating the formation of CoOOH. Interestingly, the 482 cm^{-1} E_g mode (overlapping with PF_6^- vibrational modes) initially increases in intensity (which was expected to decrease monotonically), along with the fading of the 727 cm^{-1} CO_3^{2-}/NO_3^- peak, suggesting PF_6^- intercalation. At higher potentials, the 482 cm^{-1} mode weakens, indicating partial anion deintercalation, and the 727 cm^{-1} A_{1g} vibration of PF_6^- emerges and intensifies, implying surface adsorption and accumulation of PF_6^- under an applied electric field. These results support that

PF_6^- follows a potential-driven intercalation and adsorption for CoFe LDH, consistent with the observations for NiFe LDH.

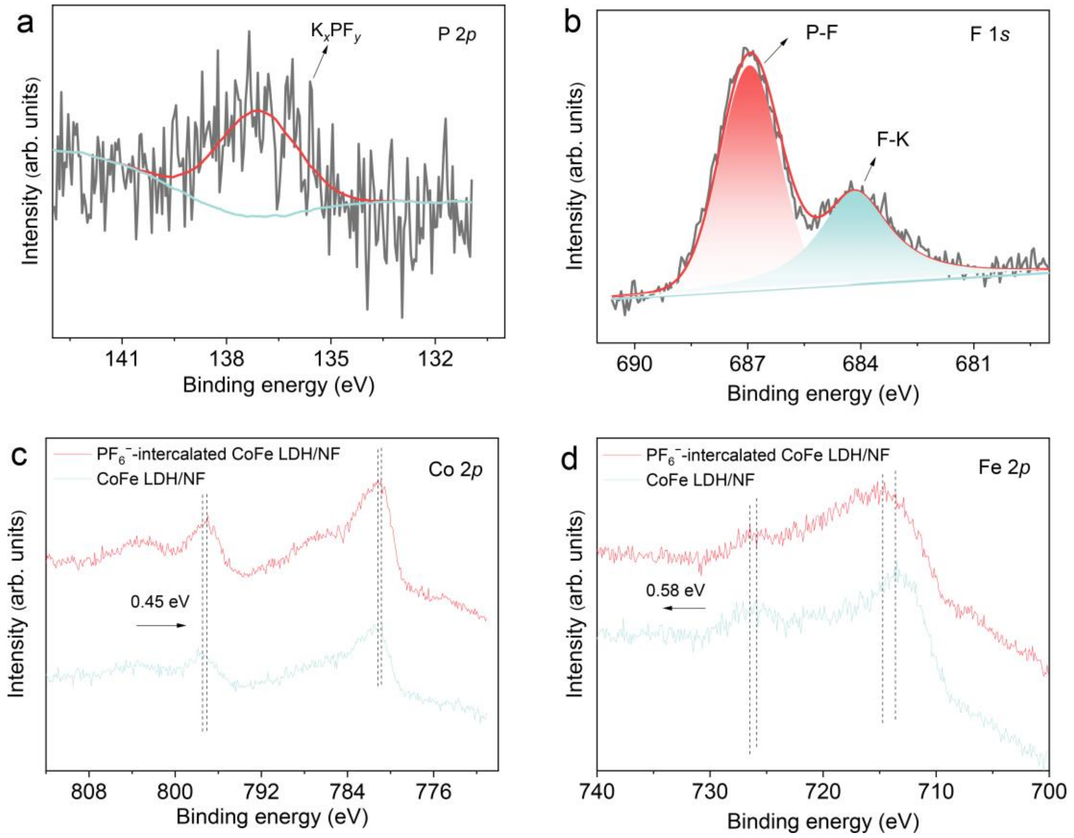

Supplementary Fig. 32 | XPS spectra. XPS spectra of PF_6^--intercalated CoFe LDH in the **(a)** P 2*p* and **(b)** F 1*s* regions. Comparison of XPS spectra of PF_6^--intercalated CoFe LDH and CoFe LDH in the **(c)** Co 2 *p* and **(d)** Fe 2*p* regions. Source data are provided as a Source Data file.

The P 2*p* and F 1*s* spectra of PF_6^--intercalated CoFe LDH (Supplementary Fig. 32a, b) exhibit similar spectral features to those observed for PF_6^--intercalated NiFe LDH, further confirming the successful incorporation of PF_6^- into the CoFe LDH. Additionally, the Co 2*p* and Fe 2*p* peaks shift toward lower and higher binding energies, respectively (Supplementary Fig. 32c, d), consistent with the electronic modulation observed in NiFe LDH.

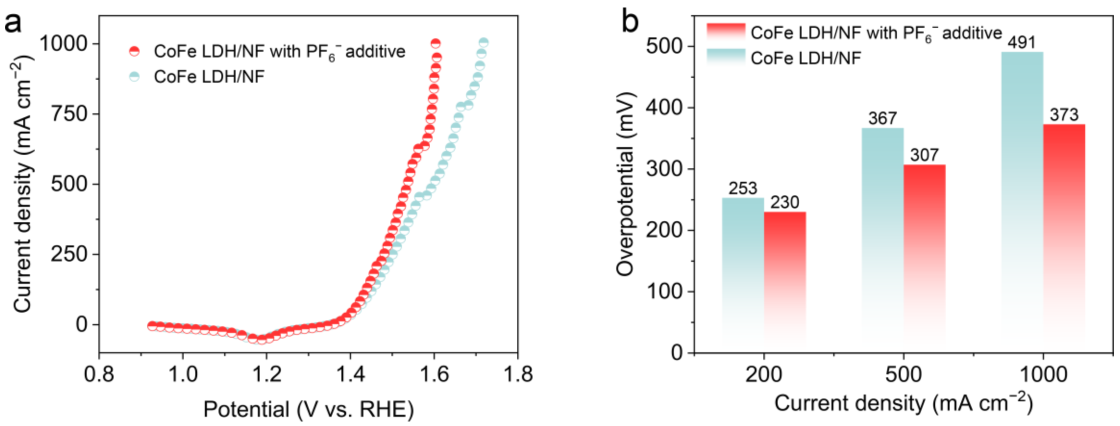

Supplementary Fig. 33 | Evaluation of activities. **(a)** Polarization curves with 100% *iR* correction and **(b)** comparison of overpotential for CoFe LDH/NF measured in PF_6^--free *versus* PF_6^--containing seawater. Source data are provided as a Source Data file.

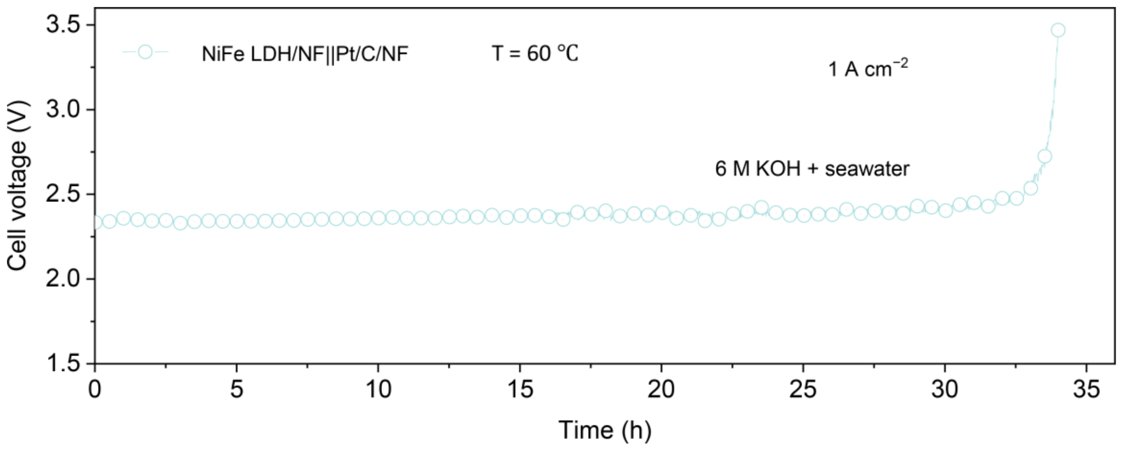

Supplementary Fig. 34 | Chronopotentiometric stability test. Chronopotentiometry curve of the NiFe LDH/NF‖Pt/C/NF electrolyzer conducted in PF_6^--free seawater at 1.0 A cm^{-2} without *iR* correction. Source data are provided as a Source Data file.

Supplementary Table 1. Elements contents of NiFe LDH determined by ICP-MS.

Element	Wt.%	Atomic%
Ni	57.84	34.71
Fe	17.52	11.05
O	24.64	54.24

Supplementary Table 2. Comparison of the overpotential of NiFe LDH/NF in PF_6^--containing seawater with reported anodic systems.

Anode	Electrolyte	*j* (mA cm^{-2})	Overpotential@*j* (mV)	Ref.
NiFe LDH/NF (with KPF_6 as electrolyte additive)	1 M KOH + seawater + 20 mM PF_6^-	1000	282	This work
		500	247	
		200	209	
NiFe LDH/NF (with Na_3PO_4 as electrolyte additive)	1 M KOH + 0.5 M NaCl+ 0.5 M Na_3PO_4	500	327	*J. Energy Chem.* **72**, 361–369 (2022)
$P_3O_{10}^{5-}$-$Ni(OH)_2$/NF	1 M KOH + seawater	400	340	*Adv. Energy Mater.* **15**, 2402883 (2025)
Ir/CoFe LDH/CP	6 M NaOH + 2.8 M NaCl	10	202	*Nat. Commun.* **15**, 1973 (2024)
PO_4^{3-}-NiFe LDH/TM	6 M KOH + Seawater	1000	330	*Nat. Commun.* **15**, 10351 (2024)
$Ni_3FeN@PO_4^{3-}$/NF	1 M KOH + seawater	1000	344	*Adv. Mater.* **37**, 2415421 (2024)
CoFePBA/Co_2P/NF	20 wt % NaOH + saturated NaCl	200	310	*Angew. Chem. Int. Ed.* **62**, e202309882 (2023)
NiFe/NiS_x/NF	6 M KOH + 1.5 M NaCl	400	396	*Proc. Natl. Acad. Sci. U. S. A.* **116**, 6624 (2019)
NiFe LDH/NF (with Na_2SO_4 as electrolyte additive)	1 M NaOH + 0.5 M NaCl + 0.05 M Na_2SO_4	400	340	*Angew. Chem. Int. Ed.* **60**, 22740–22744 (2021)
S-(Ni,Fe)OOH/NF	1 M KOH + seawater	100	300	*Energy Environ. Sci.* **13**, 3439 (2020)
		500	398	
NiFeBa LDH/Ni mesh	1 M NaOH + seawater + 0.05 M Na_2SO_4	400	298	*Adv. Mater.* **36**, 2411302 (2024)
RuMoNi/NF	1 M KOH + seawater	500	397	*Nat. Commun.* **14**, 3607 (2023)
Ni-Fe-Ce-B/MS	1 M KOH + seawater	100	271	*Appl. Catal. B Environ.* **343**, 123560 (2024)
CrO_4^{2-}-NiFe LDH/Cr_2O_3/NF	1 M KOH + seawater	1000	323	*Nat. Commun.* **15**, 6624 (2024)
NiFe LDH/NF (with CrO_4^{2-} as electrolyte additive)	1 M KOH + seawater + 0.1 M CrO_4^{2-}	100	253	*J. Colloid Interf. Sci.* **665**, 240–250 (2024)
CoFe-C_i@GQDs/NF	1 M KOH with 0.5 M NaCl	100	254	*Nat. Sustain.* **7**, 158–167 (2024)
NiFePBA/$Ni(OH)_2$/NF	1 M KOH + seawater	1000	349	*Nano Today* **58**, 102454 (2024)
WO_4^{2-}-NiFe LDH/NF	1 M KOH + seawater	1000	412	*Small* **20**, 2311431 (2024)
(NiFe)C_2O_4/NF	1 M KOH + seawater	1000	349	*Angew. Chem. Int. Ed.* **63**, e202316522 (2024)

Supplementary Table 3. Comparison of our research with key researches on anions for chloride repulsion and extended electrolysis stability.

Key anion	Anode	Durability@*j*	Electrolyte	Ref.
PF_6^- (with KPF_6 as electrolyte additive)	NiFe LDH/NF	5000 h@1000 mA cm^{-2}<br>2300 h@2000 mA cm^{-2}<br>(three-electrode system)	1 M KOH + seawater + 20 mM PF_6^-	This work
PO_4^{3-} (with Na_3PO_4 as electrolyte additive)	NiFe LDH/NF	500 h@500 mA cm^{-2}<br>(two-electrode system)	1 M KOH + 0.5 M NaCl+ 0.5 M Na_3PO_4	*J. Energy Chem*. **72**, 361–369 (2022).
$P_3O_{10}^{5-}$ ($P_3O_{10}^{5-}$ poly-oxyanion passivation layer on $Ni(OH)_2$ surface)	$P_3O_{10}^{5-}$-$Ni(OH)_2$/NF	240 h@1400 mA cm^{-2}<br>(two-electrode system)	1 M KOH + seawater	*Adv. Energy Mater.* **15**, 2402883 (2025)
PO_4^{3-} (PO_4^{3-}-intercalated NiFe LDH)	PO_4^{3-}-intercalated NiFe LDH/TM	1000 h@1000 mA cm^{-2}<br>(two-electrode system)	6 M KOH + Seawater	*Nat. Commun.* **15**, 10351 (2024)
(PO_4^{3-}) $Ni_3FeN@PO_4^{3-}$	$Ni_3FeN@PO_4^{3-}$/NF	2500 h@1000 mA cm^{-2}<br>(three-electrode system)	1 M KOH + seawater	*Adv. Mater.* **37**, 2415421 (2024)
PO_4^{3-} & $Fe(CN)_6^{3-}$ (CoFePBA/Co_2P)	CoFePBA/Co_2P/NF	1000 h@200 mA cm^{-2}<br>(three-electrode system)	20 wt % NaOH + saturated NaCl	*Angew. Chem. Int. Ed.* **62**, e202309882 (2023)
SO_4^{2-} (NiS_x layer)	NiFe/NiS_x/NF	1000 h@400 mA cm^{-2}<br>(three-electrode system)	6 M KOH + 1.5 M NaCl	*Proc. Natl. Acad. Sci. U. S. A.* **116**, 6624 (2019)
SO_4^{2-} (with Na_2SO_4 as electrolyte additive)	NiFe LDH/NF	1000 h@400 mA cm^{-2}<br>(three-electrode system)	1 M NaOH + 0.5 M NaCl + 0.05 M Na_2SO_4	*Angew. Chem. Int. Ed.* **60**, 22740–22744 (2021)
SO_4^{2-} (with SO_4^{2-} fixation layer on NiFeBa LDH)	NiFeBa LDH/Ni mesh	10000 h@400 mA cm^{-2}<br>(three-electrode system)	(1 M NaOH + seawater + 0.05 M Na_2SO_4	*Adv. Mater.* **36**, 2411302 (2024)
CO_3^{2-}& GQDs (CO_3^{2-}-intercalated CoFe LDH with GQDs anchoring)	CoFe-C_i@GQDs/NF	2800 h@1250 mA cm^{-2}<br>(three-electrode system)	1 M KOH with 0.5 M NaCl	*Nat. Sustain.* **7**, 158–167 (2024)
$C_2O_4^{2-}$ & CO_3^{2-} ((NiFe)C_2O_4)	(NiFe)C_2O_4/NF	600 h@1000 mA cm^{-2}<br>(three-electrode system)	1 M KOH + seawater	*Angew. Chem. Int. Ed.* **63**, e202316522 (2024)
MoO_4^{2-} & O_s-Cl (Os-Ni_4Mo/MoO_2)	Os-Ni_4Mo/MoO_2/NF	2500 h@500 mA cm^{-2}<br>(two-electrode system)	1 M KOH + seawater	*Adv. Mater.* **36**, 2408982 (2024)

MoO_4^{2-} (Mo leaching from RuMoNi)	RuMoNi/NF	3000 h@500 mA cm^{-2} (three-electrode system)	1 M KOH + seawater	*Nat. Commun.* **14**, 3607 (2023)
$B(OH)_4^-$ (Ni-Fe-Ce-B)	Ni-Fe-Ce-B/MS	100 h@500 mA cm^{-2} (three-electrode system)	1 M KOH + seawater	*Appl. Catal. B Environ.* **343**, 123560 (2024)
CrO_4^{2-} (CrO_4^{2-}-intercalated NiFe LDH/Cr_2O_3)	CrO_4^{2-}-NiFe LDH/Cr_2O_3/NF	1000 h@1000 mA cm^{-2} (three-electrode system)	1 M KOH + seawater	*Nat. Commun.* **15**, 6624 (2024)
CrO_4^{2-} (with CrO_4^{2-} as electrolyte additive)	NiFe LDH/NF	60 h@200 mA cm^{-2} (three-electrode system)	1 M KOH + seawater + 0.1 M CrO_4^{2-}	*J. Colloid Interf. Sci.* **665**, 240–*250 (*2024)
SeO_4^{2-} (Co-$NiSe_2$)	Co-$NiSe_2$/GC	1500 h@500 mA cm^{-2} (two-electrode system)	1 M KOH + seawater	*Appl. Catal. B Environ.* **344**, 123658 (2024)
SeO_x^- (SeO_3^- & SeO_4^{2-}) (NiS_x layer)	Se_NiFe LDH/NF	50 h@10 mA cm^{-2} (two-electrode system)	1 M KOH + 0.5 M NaCl	*Mater. Today Energy* **19**, 100575 (2021)
$Al(OH)_n^-$ (CoFeAl LDH)	CoFeAl LDH/NF	500 h@1000 mA cm^{-2} (three-electrode system)	20wt.% NaOH + satu. NaCl	*Nat. Commun.* **15**, 4712 (2024)
$Fe(CN)_6^{3-}$ (NiFePBA)	NiFePBA/$Ni(OH)_2$/NF	1000 h@1000 mA cm^{-2} (three-electrode system)	1 M KOH + seawater	*Nano Today* **58**, 102454 (2024)
WO_4^{2-} (WO_4^{2-}-intercalated NiFe LDH)	WO_4^{2-}-NiFe LDH/NF	350 h@1000 mA cm^{-2} (three-electrode system)	1 M KOH + seawater	*Small* **20**, 2311431 (2024)

Supplementary Table 4. Elements contents of PF_6^--intercalated NiFe LDH determined by ICP-MS.

**Element**	**wt.%**	**Atomic%**
Ni	56.73	34.65
Fe	17.93	11.51
O	19.40	43.46
F	4.81	9.08
P	1.12	1.3

Supplementary Table 5. Comparison of the overpotentials of CoFe LDH/NF anode in PF_6^--containing seawater with recently reported CoFe-based anodes.

Anodes	Electrolyte	*j* (mA cm^{-2})	Overpotential (mV)	Reference
CoFe LDH/NF (with KPF_6 as electrolyte additive)	1 M KOH + seawater + 20 mM PF_6^-	200	230	This work
		500	307	
		1000	373	
CF@CF-phy/NF	1 M KOH +seawater	500	330	*ACS Nano* **19***,* 1530–1546 (2025)
CoFeAl LDH/NF	20wt.% NaOH + satu. NaCl	10	256	*Nat. Commun.* **15**, 4712 (2024)
		200	~320	
CoFe-Ci@GQD	1 M KOH + 0.5 M NaCl	100	255	*Nat. Sustain.* **7**, 158–167 (2024)
Ir/CoFe LDH	6 M NaOH + 2.8 M NaCl	10	202	*Nat. Commun.* **15** 1973 (2024)
Cr-CoFe LDH/NF	1 M KOH + seawater	500	334	*Small* **20**, 2307294 (2024)
B-Co_2Fe LDH/NF	1 M KOH + seawater	100	310	*Nano Energy* **83**, 105838 (2021)
		500	376	
$CoCO_3$/CoFe LDH/NF	1 M KOH + seawater	500	316	*Small* **21**, 2409627 (2025)
RuCo-$CoFe_2O_4$@IF	1 M KOH + seawater	1000	425	*Chem. Eng. J.* **503**, 158346 (2025)

Supplementary Table 6. Comparison of the stability of CoFe LDH/NF anode in PF_6^--containing seawater with recently reported CoFe-based anodes.

**Anodes**	**Electrolyte**	***j* (mA cm^{-2})**	**Stability (h)**	**Reference**
CoFe LDH/NF (with KPF_6 as electrolyte additive)	1 M KOH + seawater + 20 mM PF_6^-	2000	1200	This work
CF@CF-phy/NF	1 M KOH +seawater	1000	1000	*ACS Nano* **19***,* 1530–1546 (2025)
CoFeAl LDH/NF	20wt.% NaOH + satu. NaCl	1000	500	*Nat. Commun.* **15**, 4712 (2024)
		2000	350	
CoFe-Ci@GQD	1 M KOH + 0.5 M NaCl	1250	2800	*Nat. Sustain.* **7**, 158–167 (2024)
Ir/CoFe LDH	6 M NaOH + 2.8 M NaCl	800	1000	*Nat. Commun.* **15** 1973 (2024)
Cr-CoFe LDH/NF	1 M KOH + seawater	500	100	*Small* **20**, 2307294 (2024)
B-Co_2Fe LDH/NF	1 M KOH + seawater	500	100	*Nano Energy* **83**, 105838 (2021)
CeO_{2-x}@CoFe LDH/NF	1 M KOH + 0.5 M NaCl	50	35	*Inorg. Chem. Front*. **7**, 4461–4468 (2020)
$CoCO_3$/CoFe LDH/NF	1 M KOH + seawater	1000	1000	*Small* **21**, 2409627 (2025)
CoFePBA/Co_2P	20wt.% NaOH + satu. NaCl	1000	1000	*Angew. Chem. Int. Ed.* **62**, e202309882 (2023)
		2000	100	
CoFe-Ni_2P/NF	1 M KOH + seawater	500	500	*Adv. Energy Mater.* **13**, 2301475 (2023)
FCDs/FeCoSe-VSe/NF	1 M KOH + seawater	200	200	*Appl. Surf. Sci.* **680**, 161456 (2025)
RuCo-$CoFe_2O_4$@IF	1 M KOH + seawater	1000	150	*Chem. Eng. J.* **503**, 158346 (2025)

Supplementary Table 7. Comparison of the extended electrolysis stability achieved by the PF_6^--boosted ASO strategy with existing Cl^--repelling strategy (surface chloride immobilization/electrostatic repulsion/physical shielding).

Strategy	Anode	Durability@*j*	Electrolyte	Ref.
electrostatic repulsion	NiFe LDH/NF	5000 h@1000 mA cm^{-2}<br><br>2300 h@2000 mA cm^{-2}	1 M KOH + seawater + 20 mM PF_6^-	This work
surface chloride immobilization (AgCl)	NiFe LDH@Ag/NF	5000 h@400 mA cm^{-2}	6 M KOH + Seawater	*Adv. Mater.* **36**, 2306062 (2024)
surface chloride immobilization (IrCl)	Ir/CoFe LDH	1000 h@800 mA cm^{-2}	6 M NaOH + 2.8 M NaCl	*Nat. Commun.* **15**, 1973 (2024)
surface chloride immobilization & electrostatic repulsion (MoO_4^{2-} & O_s-Cl)	Os-Ni_4Mo/MoO_2/NF	2500 h@500 mA cm^{-2}	1 M KOH + seawater	*Adv. Mater.* **36**, 2408982 (2024)
electrostatic repulsion (PO_4^{3-})	PO_4^{3-}-intercalated NiFe LDH/NF	1000 h@1000 mA cm^{-2}	6 M KOH + Seawater	*Nat. Commun.* **15**, 10351 (2024)
electrostatic repulsion (SO_4^{2-})	NiFe/NiS_x/NF	1000 h@400 mA cm^{-2}	6 M KOH + 1.5 M NaCl	*Proc. Natl. Acad. Sci. U. S. A.* **116**, 6624 (2019)
electrostatic repulsion (SO_4^{2-})	Pt/NiFe LDH/NF	1000 h@400 mA cm^{-2}	1 M NaOH + 0.5 M NaCl + 0.05 M Na_2SO_4	*Angew. Chem. Int. Ed.* **60**, 22740–22744 (2021)
electrostatic repulsion (SO_4^{2-} immobilization through Ba^{2+})	NiFeBa LDH/Ni mesh	10000 h@400 mA cm^{-2}	(1 M NaOH + seawater + 0.05 M Na_2SO_4	*Adv. Mater.* **36**, 2411302 (2024)
electrostatic repulsion (CO_3^{2-}& GQDs)	CoFe-C_i@GQDs/NF	2800 h@1250 mA cm^{-2}	1 M KOH + 0.5 M NaCl	*Nat. Sustain.* **7**, 158–167 *(2024)*
physical shielding (MnO_x)	MnO_x/NiFe LDH/NF	72 h@50 mA cm^{-2}	1 M KOH + seawater	*Inorg. Chem.* **61**, 15256 (2021)
physical shielding (CeO_2)	CeO_2-NiFe LDH/NF	500 h@1000 mA cm^{-2}	1 M KOH + seawater	*J. Energy. Chem.* **91**, 306 (2024)
physical shielding (MoO_3)	MoO_3@CoO/CC	350 h@1000 mA cm^{-2}	1 M KOH + seawater	*Small* **20**, 2311431 (2024)

Supplementary Table 8. Comparison of cell voltage and tolerance of NiFe LDH/NF‖Pt/C/NF with reported two-electrode electrolyzers.

**Anode‖cathode**	**Tolerance@*j***	**Tolerance@cell voltage**	**Electrolyte**	**Ref.**
NiFe LDH/NF‖Pt/C/NF	1000 h@1000 mA cm^{-2}	1000 h@2.02 V	6 M KOH + seawater + 20 mM PF_6^-	This work
NiFe LDH/NF‖Pt/NF	1000 h@400 mA cm^{-2}	1000 h@~2.2 V	6 M NaOH + seawater + 0.23 M Na_2SO_4	*Angew. Chem. Int. Ed.* **133**, 22922 (2021)
Ce-NiFe LDH/NF‖Pt/C/NF	120 h@300 mA cm^{-2}	120 h@~2.24 V	1 M KOH + seawater	*J. Energy. Chem.* **91**, 306 (2024)
NiFeBa LDH/Ni mesh‖Raney Ni	100 h@400 mA cm^{-2}	100 h@~1.98 V	6 M NaOH + seawater + 0.23 M Na_2SO_4	*Adv. Mater.* **36**, 2411302 (2024)
$(NiFe)C_2O_4$/NF‖Pt/C/NF	150 h@500 mA cm^{-2}	150 h@~2.4 V	1 M KOH + seawater	*Angew. Chem. Int. Ed.* **63**, e202316522 (2024)
CoFeAl LDH/NF‖NF	500 h@1000 mA cm^{-2}	500 h@~2.06 V	20wt.% NaOH + satu. NaCl	*Nat. Commun.* **15**, 4712 (2024)
NiFePBA/$Ni(OH)_2$/NF‖Pt/C/NF	500 h@500 mA cm^{-2}	500 h@~2.23 V	1 M KOH + seawater	*Nano Today* **58**, 102454 (2024)
Os-Ni_4Mo/MoO_2/NF (+, -)	200 h@200 mA cm^{-2}	200 h@~2.3 V	1 M KOH + seawater	*Adv. Mater.* **36**, 2408982 (2024)
PO_4^{3-}-intercalated NiFe LDH/NF‖Raney Ni	200 h@500 mA cm^{-2}	200 h@~2.08 V	6 M KOH + seawater	*Nat. Commun.* **15**, 10351 (2024)
CoFe-Ci@GQDs/NF (+, -)	200 h@442 mA cm^{-2}	200 h@~2.71 V	1 M KOH with 0.5 M NaCl	*Nat. Sustain.* **7**, 158–167 *(2024)*
NiFe LDH@Ag/NF‖Cu_2S@Ni	1200 h@400 mA cm^{-2}	200 h@~1.98 V	1 M KOH + seawater	*Adv. Mater.* **36**, 2306062 (2024)
MoO_3@CoO/CC (+, -)	500 h@1000 mA cm^{-2}	500 h@~1.93 V	1 M KOH + seawater	*Nat. Commun.* **15**, 2481 (2024)
$Ni_3FeN@PO_4^{3-}$/NF‖Pt-Ni@NiMoN/NF	140 h@1000 mA cm^{-2}	140 h@~2.07 V	1 M KOH + seawater	*Adv. Mater.* **37**, 2415421 (2024)
$P_3O_{10}^{5-}$-$Ni(OH)_2$/NF‖Pt/C/NF	240 h@1400 mA cm^{-2}	240 h~2.2V	1 M KOH + seawater	*Adv. Energy Mater.* **15**, 2402883 (2025)
